# Deep Learning-Enhanced Nanozyme-Based Biosensors for Next-Generation Medical Diagnostics

**DOI:** 10.3390/bios15090571

**Published:** 2025-09-01

**Authors:** Seungah Lee, Nayra A. M. Moussa, Seong Ho Kang

**Affiliations:** 1 Department of Applied Chemistry and Institute of Natural Sciences, Kyung Hee University, Yongin-si 17104, Gyeonggi-do, Republic of Korea; salee@khu.ac.kr; 2 Department of Chemistry, Graduate School, Kyung Hee University, Yongin-si 17104, Gyeonggi-do, Republic of Korea; nayra.moussa@khu.ac.kr

**Keywords:** nanozymes, deep learning, biosensing, smartphone-based detection, point-of-care diagnostics

## Abstract

The integration of deep learning (DL) and nanozyme-based biosensing has emerged as a transformative strategy for next-generation medical diagnostics. This review explores how DL architectures enhance nanozyme design, functional optimization, and predictive modeling by elucidating catalytic mechanisms such as dual-atom active sites and substrate-surface interactions. Key applications include disease biomarker detection, medical imaging enhancement, and point-of-care diagnostics aligned with the ASSURED criteria. In clinical contexts, advances such as wearable biosensors and smart diagnostic platforms leverage DL for real-time signal processing, pattern recognition, and adaptive decision-making. Despite significant progress, challenges remain—particularly the need for standardized biomedical datasets, improved model robustness across diverse populations, and the clinical translation of artificial intelligence (AI)-enhanced nanozyme systems. Future directions include integration with the Internet of Medical Things, personalized medicine frameworks, and sustainable sensor development. The convergence of nanozymes and DL offers unprecedented opportunities to advance intelligent biosensing and reshape precision diagnostics in healthcare.

## 1. Introduction

Nanozyme-engineered nanomaterials—synthetic constructs that mimic the catalytic activity of natural enzymes—have emerged as promising platforms for biosensing applications in recent years [1,2,3]. Compared to biological enzymes, nanozymes offer superior chemical stability, lower production costs, and greater scalability, making them well suited for point-of-care (POC) diagnostics and clinical testing [4,5,6]. Their distinctive physicochemical properties, such as tunable surface chemistry, multivalent active sites, and resistance to harsh conditions, further support their application in robust sensing across diverse environments [7]. For clarity, although nanozymes are synthetic and not of biological origin, this review adopts the conventional usage of the term “biosensor,” which refers to platforms detecting biological targets irrespective of whether the recognition or catalytic element is biological or synthetic [5,8,9]. Despite these advantages, the clinical translation of nanozyme-based biosensors remains limited. Key challenges include an incomplete understanding of nanozyme catalytic mechanisms at the atomic level, difficulty in tuning activity and substrate specificity, and the lack of standardized protocols for performance optimization and validation [10].

Recent advances in artificial intelligence (AI), particularly deep learning (DL), offer effective strategies to address these limitations. DL models, such as convolutional neural networks (CNNs), can process large, complex spectroscopic, microscopic, and kinetic datasets without manual feature extraction [11,12,13,14,15,16]. These models enable accurate prediction of key kinetic parameters, including Michaelis–Menten constants (*K*_m_) and maximum reaction rates (*V*_max_), and can inform the rational design of nanozymes for specific sensing applications [17,18,19].

Integrating DL with nanozyme-based biosensors has shown considerable promise in medical diagnostics. AI-enhanced nanozyme platforms have achieved high sensitivity and specificity in detecting clinically relevant biomarkers, such as nucleic acids, proteins, and metabolites, associated with infectious diseases, cancer, and metabolic disorders [20,21,22]. These advancements support the development of portable and wearable biosensors capable of real-time analysis, aligned with the World Health Organization’s ASSURED criteria (Affordable, Sensitive, Specific, User-friendly, Rapid and robust, Equipment-free, and Deliverable), and contribute to the advancement of personalized health monitoring [23,24].

Despite recent progress, several challenges persist. The lack of high-quality, standardized datasets limits the reproducibility and generalizability of AI models in biological and clinical contexts [25]. In addition, the scalability of DL-guided nanozyme synthesis and the interpretability of model predictions remain open research questions. Overcoming these barriers will require interdisciplinary collaboration among chemists, materials scientists, computer scientists, and clinicians, along with a shared commitment to data sharing and methodological standardization.

The convergence of AI-driven biosensors and nanozyme-enabled sensing is particularly timely in the post-COVID-19 era. The pandemic not only exposed the limitations of centralized laboratory testing but also accelerated the adoption of mobile health technologies, telemedicine, and Internet of Things (IoT)-based diagnostics. Within this shifting landscape, nanozyme-based biosensors offer low-cost, robust, and portable assays, while DL models deployed on smartphones provide automated interpretation of complex biosensing signals. Furthermore, IoT integration facilitates real-time data sharing for population-level surveillance, early outbreak detection, and personalized health monitoring. Together, these advances position DL-nanozyme biosensors as strategically aligned with global healthcare priorities, underscoring their potential to shape the future trajectory of decentralized diagnostics [26,27].

In this regard, the present moment offers a particularly opportune time for synthesizing this field. On the nanozyme side, advances such as single- and dual-atom engineering, defect modulation, and surface functionalization have markedly improved catalytic activity, selectivity, and stability. In parallel, DL architectures—including CNNs, YOLO, and MobileNet—have demonstrated powerful capabilities in automated feature extraction, real-time signal interpretation, and multiplexed data analysis. These two domains are now converging, with several proof-of-concept demonstrations highlighting their combined potential for intelligent biosensing. As such, a comprehensive review is timely to consolidate current knowledge, identify persisting challenges, and chart future directions for DL-enhanced nanozyme-based biosensors.

## 2. Fundamentals of Nanozymes for Biosensing

### 2.1. Definition and Classification of Nanozymes

Nanozymes are a class of nanomaterials—typically 1–100 nm in size—that exhibit intrinsic enzyme-like catalytic activity [10,28,29]. Unlike natural enzymes, which are protein-based and prone to denaturation under harsh conditions, nanozymes consist of inorganic or hybrid organic-inorganic materials engineered to replicate the catalytic behavior of enzymes such as peroxidases, oxidases, catalases, and superoxide dismutases [30]. Their catalytic efficiency is driven by key physicochemical properties, including high surface-to-volume ratios, tunable surface chemistries, and structural robustness, enabling function under extreme pH, temperature, and proteolytic stress—conditions under which natural enzymes typically lose activity [31,32,33].

Nanozymes are commonly classified by either their composition or the type of enzyme activity they mimic [34]. Table 1 provides an overview of major nanozyme categories, representative materials, associated enzyme-mimetic functions, and typical application areas.

The most widely studied nanozymes are metal- and metal oxide-based, including nanoparticles of noble metals (e.g., Au, Ag, Pt, and Pd), transition metal oxides (e.g., Fe_3_O_4_, MnO_2_, CuO, CeO_2_, and Co_3_O_4_), and their bimetallic or alloyed derivatives [35,36,37]. These materials typically exhibit peroxidase-, oxidase-, or catalase-like activities. For instance, Fe_3_O_4_ nanoparticles mimic peroxidase activity by catalyzing the decomposition of H_2_O_2_, whereas CeO_2_ nanozymes demonstrate redox-dependent oxidase and catalase activity [34,35,36,38,39,40].

Carbon-based nanozymes, such as graphene, graphene oxide, carbon nanotubes, carbon quantum dots, and graphitic carbon nitride, offer high electronic conductivity and tunable surface functionality, making them well suited for biosensing and electrocatalytic applications [41,42,43].

With their high porosity, crystallinity, and modular architecture, metal-organic frameworks (MOFs) and covalent organic frameworks have emerged as versatile platforms for incorporating enzyme-mimetic sites [44,45,46].

Polymeric and organic nanozymes, though less widely studied, have demonstrated biomimetic activity by replicating features of native enzyme microenvironments—such as hydrogen-bonding networks and hydrophobic domains—offering enhanced selectivity and biocompatibility in biological matrices [47].

**Figure 1:**
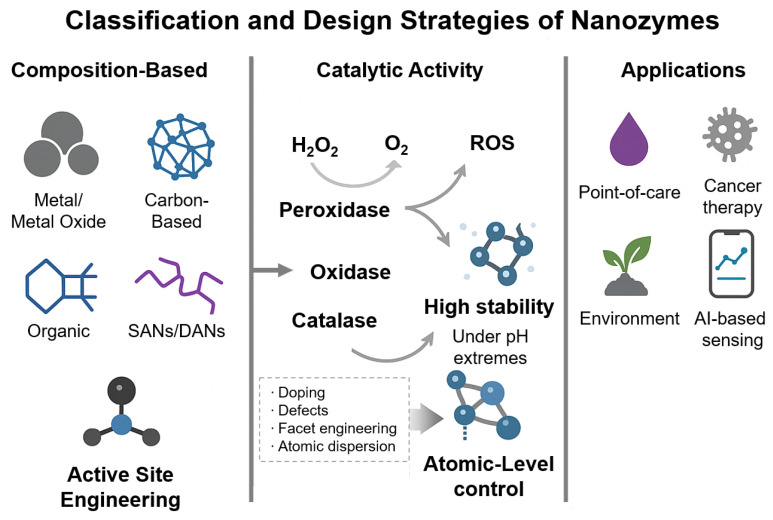
Overview of nanozyme types and design strategies. The figure illustrates composition-based categories, representative catalytic activities, common applications, and active site engineering methods such as doping, defects, and atomic dispersion. Partially generated with ChatGPT (OpenAI, 2025).

Single-atom nanozymes (SANs) and dual-atom nanozymes (DANs) represent a cutting-edge direction in nanozyme design. In these systems, isolated metal atoms or metal pairs are precisely anchored onto support surfaces, enabling high turnover frequencies, exceptional selectivity, and atomic-level control over active site architecture [48,49]. Figure 1 schematically illustrates the design strategies and functional classifications of nanozymes, highlighting major composition-based categories, catalytic activities (e.g., peroxidase, oxidase), and advanced engineering approaches such as heteroatom doping, defect creation, and atomic-level modulation for biomedical applications.

Recent advances in active site engineering—including heteroatom doping, defect generation, and facet modulation—have significantly improved nanozyme performance by enhancing substrate selectivity and reaction kinetics. These strategies aim to bridge the specificity gap between nanozymes and natural enzymes. Increasing attention is also being paid to biocompatibility and cytotoxicity, especially for in vivo applications. Although many metal-based nanozymes offer good chemical stability, their long-term fate, biodegradability, and potential accumulation in biological systems remain under investigation. This has spurred interest in organic, carbon-based, and surface-modified nanozymes as safer alternatives for clinical use.

While this review focuses on biosensing, nanozymes have also been explored in cancer therapy (e.g., chemodynamic therapy), antimicrobial treatments, environmental pollutant degradation, and reactive oxygen species (ROS) modulation, highlighting their broad functional potential.

Finally, as nanozyme architectures grow increasingly complex, AI-driven methods—particularly DL—provide a powerful data-driven framework to elucidate structure-activity relationships and accelerate the rational design of nanozymes for biomedical applications.

### 2.2. Catalytic Mechanisms and Active Site Engineering

The exceptional catalytic efficiency of nanozymes, which mimic natural enzymes, arises from their unique physicochemical properties at the nanoscale [50,51]. A comprehensive understanding of their underlying catalytic mechanisms is essential for rational design and performance optimization. Active site engineering offers a powerful strategy to enhance nanozyme specificity, activity, and stability, thereby expanding their application potential. The following sections examine the fundamental catalytic mechanisms (Section 2.2.1) and key active site engineering strategies (Section 2.2.2), both critical to advancing nanozyme-based biosensor performance.

#### 2.2.1. Catalytic Mechanisms of Nanozymes

**Figure 2:**
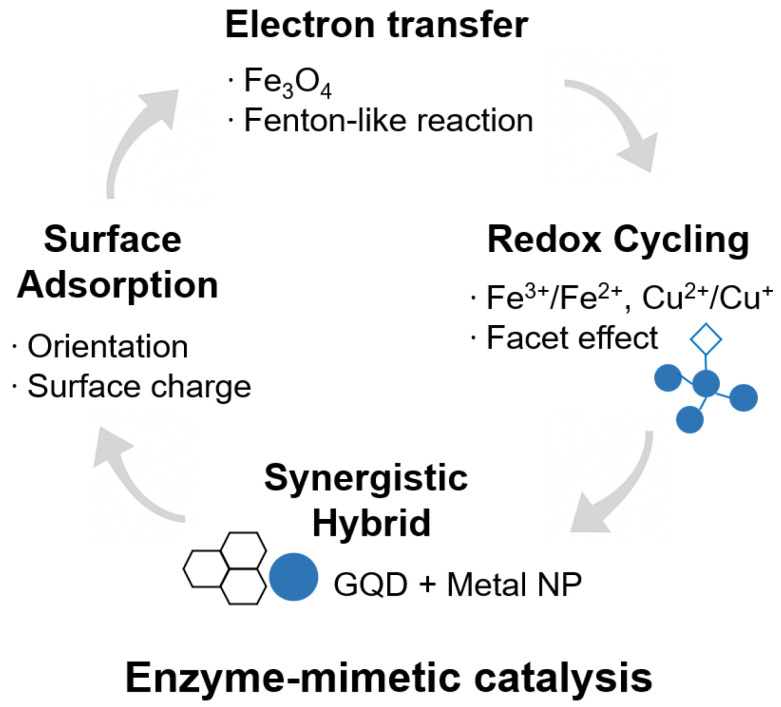
Schematic of major catalytic mechanisms of nanozymes.

Nanozyme catalytic activity arises from a combination of nanoscale physicochemical processes, including electron transfer, surface adsorption, redox cycling, and synergistic interactions in hybrid systems [52,53,54,55] (Figure 2). While functionally analogous to the mechanisms of natural enzymes, these processes are governed by distinct features—such as surface properties, electronic structures, and nano-confinement effects—that critically influence substrate binding and reaction kinetics.

(1) Electron Transfer Reactions: Electron transfer is a primary catalytic mechanism in many nanozymes, particularly those based on transition metal oxides (e.g., Fe_3_O_4_ and MnO_2_) and noble metals (e.g., Au, Pt) [37,56,57,58]. For instance, Fe_3_O_4_ nanozymes catalyze H_2_O_2_ decomposition via a Fenton-like reaction, producing reactive hydroxyl radicals (‧OH) through redox cycling between Fe^3+^ and Fe^2+^ [58]. Their high surface area and abundance of active sites facilitate efficient electron exchange with substrates. Similarly, noble metal nanozymes act as electron reservoirs, enabling charge redistribution and lowering activation energy during catalytic transformations [59]. While electron transfer underlies redox activity, catalytic efficiency also depends on the spatial proximity between substrates and active sites.

(2) Surface Adsorption and Orientation: Nanozymes exhibit high surface-to-volume ratios and tunable surface chemistries that facilitate efficient substrate adsorption and favorable molecular orientation [60,61]. This spatial arrangement increases local substrate concentration and aligns reactive groups in geometries conducive to catalysis. Surface properties, such as charge, hydrophobicity, and functional group availability, strongly influence binding affinity and reaction specificity. Alongside adsorption effects, redox cycling supports sustained catalytic turnover by enabling continuous electron transfer during the reaction process.

(3) Redox Cycling and Valence Modulation: In metal-based nanozymes, catalytic activity is often driven by dynamic redox cycling, where metal ions (Fe^3+^/Fe^2+^, Cu^+^/Cu^2+^) alternate between oxidation states to sustain continuous electron flow during catalytic turnover [62,63,64,65,66]. The exposed crystal facets of nanozymes can stabilize specific valence states, thereby modulating electron transfer kinetics and enhancing catalytic efficiency. Moreover, incorporating complementary materials into hybrid nanozyme architectures can further improve performance by introducing synergistic effects and broadening functional capabilities.

(4) Synergistic Effects in Hybrid Nanozymes: Hybrid nanozymes—comprising multiple nanomaterials such as graphene quantum dots combined with metal nanoparticles—often exhibit synergistic effects that exceed the catalytic performance of individual components [67,68,69,70,71,72]. In photoactive hybrids, for instance, light irradiation generates electron-hole pairs in semiconductor domains, followed by charge transfer to metal sites, thereby enhancing redox activity. These synergistic interactions expand reaction pathways, improve electron transport, and enable broader functional performance.

#### 2.2.2. Active Site Engineering for Improved Performance

Active site engineering is a key strategy for precisely tuning the catalytic behavior of nanozymes by manipulating their atomic and molecular environments [72,73,74,75,76]. Core approaches include doping, defect formation, facet exposure, atomic dispersion, and surface functionalization (summarized in Table 2). These strategies aim to narrow the performance gap between artificial and natural enzymes by enhancing catalytic efficiency, substrate selectivity, and operational stability.

Surface doping and elemental substitution are widely adopted methods that involve the intentional incorporation of heteroatoms (e.g., N, P, and S) into the lattice of a host material or the replacement of existing atoms to modify the electronic structure and surface properties [77,78,79,80]. These alterations can create or enhance active sites by modulating the electron density, introducing localized defects, or shifting the *d*-band center of surface atoms, thereby influencing substrate binding energy and reaction kinetics. For example, nitrogen-doped carbon-based nanozymes exhibit improved activity in oxygen reduction reactions (ORRs), whereas doped metal oxide nanozymes exhibit enhanced substrate affinity and turnover through electronic modulation [81,82,83,84].

Defect engineering is another critical strategy that focuses on the deliberate generation of structural imperfections such as oxygen vacancies (OVs), dislocations, and edge defects, particularly in metal oxides and two-dimensional (2D) materials [85,86,87,88,89]. These defects typically act as unsaturated coordination centers that enhance substrate adsorption and electron transfer. A prominent example is cerium oxide (CeO_2_) nanozymes, where OVs significantly boost catalase-like activity by increasing H_2_O_2_ accessibility at reactive sites [90,91]. Facet exposure engineering enables the selective presentation of catalytically active crystal planes (e.g., {111} and {100}), each with distinct surface atom arrangements and electronic characteristics that modulate catalytic behavior [91,92,93,94].

The development of SANs and DANs, where isolated metal atoms or atomic pairs are dispersed on conductive supports such as carbon frameworks and metal oxides, is a rapidly advancing frontier in nanozyme design [95,96,97,98,99]. These atomically precise configurations maximize catalytic atom utilization and typically exhibit unique reactivity because of quantum confinement effects and strong metal-support interactions. Furthermore, the electronic state and local coordination environment of single atoms can be rationally adjusted to fine-tune catalytic specificity and reaction kinetics at an unprecedented level.

In addition to structural engineering, surface modification and functionalization offer versatile strategies to enhance nanozyme-based biocompatibility and targeting specificity [100,101,102]. The covalent or non-covalent attachment of functional groups, polymers, or biological ligands (e.g., aptamers, antibodies) can direct nanozyme activity toward specific analytes, improve stability in physiological environments, and minimize non-specific interactions. Modulating surface hydrophilicity, charge, and steric hindrance further refines the local reaction microenvironment, thereby enhancing biosensing performance.

By combining strategies such as doping, defect creation, facet control, atomic dispersion, and surface functionalization, researchers are advancing the rational design of nanozymes with enzyme-like precision. These engineered platforms are achieving enhanced sensitivity, selectivity, and adaptability, expanding their utility across a wide range of diagnostic and therapeutic applications.

### 2.3. Advantages and Limitations of Nanozymes in Biosensing

The increasing interest in nanozymes for medical diagnostics is due to their unique advantages over natural enzymes; however, acknowledging their current limitations is also important. A balanced perspective on these aspects is crucial for guiding future research and development in nanozyme-enhanced biosensing.

#### 2.3.1. Advantages of Nanozymes in Biosensing

Nanozymes offer several advantages that make them highly attractive for biosensing applications [8,9,103,104]. First, unlike natural enzymes that require complex and costly purification from biological sources, nanozymes are economically feasible and scalable, enabling large-scale production. Second, they exhibit exceptional stability under harsh conditions, including extreme pH, elevated temperatures, and exposure to proteases or organic solvents, thereby extending shelf life and ensuring reliable performance in diverse biological environments where natural enzymes often denature. Third, nanozymes are synthetically tunable; their catalytic activity, substrate selectivity, and surface properties can be precisely engineered through doping, defect modulation, and surface functionalization. This level of control enables the design of nanozymes tailored to specific analytes and sensing contexts, surpassing the fixed catalytic behavior of natural enzymes.

In addition, nanozymes can be seamlessly integrated into various detection platforms, including electrochemical, optical, colorimetric, and fluorescence-based systems. Their nanoscale dimensions enable device miniaturization and facilitate incorporation into portable, POC diagnostic tools, thereby expanding their clinical utility.

#### 2.3.2. Limitations of Nanozymes in Biosensing

Despite their advantages, nanozymes face several limitations that warrant continued innovation [5,105,106]. First, their catalytic efficiency and substrate specificity generally fall short of those of natural enzymes, largely due to the absence of a well-defined protein scaffold and evolutionarily optimized active site microenvironments. While advances in active site engineering and bio-hybrid designs are narrowing this gap, fully replicating the substrate affinity and turnover rates of natural enzymes remains a significant challenge. Second, batch-to-batch variability in synthesis—arising from inconsistencies in nanoparticle size, morphology, surface defect density, and composition—can compromise reproducibility. Such variability complicates standardization, hinders quality control, and poses barriers to commercialization and clinical translation. Third, cytotoxicity remains a concern, particularly for in vivo applications. The biological safety of nanozymes depends on factors such as material composition, concentration, degradation profile, and long-term accumulation, all of which must be rigorously assessed—especially for clinical biosensors involving direct tissue contact. Fourth, interference from complex biological matrices (e.g., blood, urine, serum) can compromise sensor accuracy. Non-specific adsorption of proteins or other biomolecules may block active sites, generate false signals, or reduce detection sensitivity. Finally, although multiplexed detection is a growing area of research, achieving reliable simultaneous detection of multiple analytes with nanozyme-based systems remains technically challenging. Signal overlap, cross-reactivity, and difficulties in platform integration continue to limit the practical implementation of high-fidelity multiplexed biosensing.

Overcoming these limitations is essential to realizing the full potential of nanozymes as next-generation diagnostic tools. Future research should prioritize improved synthetic control, enhanced biocompatibility, and the integration of machine learning (ML) to enable robust, selective, and clinically viable biosensing platforms.

## 3. DL Architectures in Nanozyme Research

### 3.1. Overview of DL and CNNs

DL, a powerful subset of ML, has transformed various scientific domains by enabling computational models to learn hierarchical representations of data through multiple layers of nonlinear processing [107,108,109,110,111,112,113,114]. Unlike conventional ML approaches that heavily depend on handcrafted features, DL algorithms can autonomously extract complex patterns directly from raw, high-dimensional data. This characteristic is particularly advantageous in biosensing, where large-scale, noisy, and multivariate signals are typically contained in data.

#### 3.1.1. Understanding the DL Approach

The core of a DL approach lies in its ability to automatically extract complex, hierarchical features from raw data through a layered network of artificial neurons. Unlike traditional machine learning, which often requires manual feature engineering, DL models learn to represent data at multiple levels of abstraction. For example, in a colorimetric nanozyme-based biosensor, the model does not just analyze simple features like average color intensity. Instead, it learns to identify intricate spatial patterns, subtle color variations, and textural information from the image data, which are then used for accurate analyte detection. This process allows the model to uncover subtle, non-linear relationships within the data that are often imperceptible to human analysis or conventional algorithms.

#### 3.1.2. Signal Collection, Filtering, and Elaboration

The successful application of DL begins with the careful collection and preparation of signals. In the context of nanozyme-based biosensing, signals are typically collected as high-dimensional data, such as images, spectroscopic data, or time-series electrical signals. These raw signals are then subjected to a crucial preprocessing pipeline. This workflow includes steps such as signal filtering to remove noise and unwanted artifacts, normalization to scale data to a uniform range, and data augmentation to artificially increase the size and diversity of the training dataset. For instance, in an image-based system, augmentation techniques like rotation, scaling, or adjusting brightness are applied to ensure the model is robust to variations in lighting conditions or sensor placement. This meticulous elaboration of the signal is essential to create a clean, standardized input that enables the DL model to learn effectively and generalize across different conditions.

#### 3.1.3. Model Training and Application

Once the data is prepared, the DL model is trained on a labeled dataset. During the training phase, the model’s internal parameters (weights and biases) are iteratively adjusted to minimize the difference between its predictions and the actual known values (the labels). This optimization process, often performed using algorithms like stochastic gradient descent, fine-tunes the network to accurately map input signals to desired outputs, such as analyte concentration or the presence of a specific biomarker. After training, the model is ready for real-world application, a stage known as inference. The trained model is then deployed to new, unseen samples, where it processes the raw, pre-processed signals and provides a fast and reliable output. This final stage demonstrates the model’s ability to act as a powerful and automated tool for quantitative analysis or classification in POC and laboratory settings.

At the core of DL are artificial neural networks consisting of interconnected layers of neurons. As data propagate through these layers, the network progressively learns abstract and task-relevant features, enabling it to model complex, nonlinear relationships that conventional models often fail to capture.

Among DL architectures, CNNs are particularly effective for analyzing data with spatial or sequential structure—such as images, time-series signals, and spectroscopic data—which are common in nanozyme-based biosensing applications [115,116,117,118]. A typical CNN comprises three main components: convolutional layers for feature extraction, pooling layers for dimensionality reduction, and fully connected layers for classification or regression.

(1) Convolutional layers apply learnable filters (kernels) that scan across the input to detect localized patterns, such as edges, textures, or spectral peaks. Each filter captures a specific feature, and multiple filters collectively learn a diverse set of representations relevant to the task.

(2) Pooling layers, such as max pooling and average pooling, reduce the dimensionality of feature maps by summarizing local regions, thereby decreasing computational complexity, enhancing generalization, and improving robustness to minor spatial or temporal variations in the input.

(3) Fully connected layers integrate high-level features extracted by preceding layers to produce final outputs, such as classification labels or regression values.

The hierarchical architecture of CNNs enables early layers to capture low-level features, while deeper layers integrate these into higher-level abstractions. This layered feature extraction makes CNNs particularly well suited for analyzing complex biological signals commonly encountered in nanozyme-based sensing platforms.

### 3.2. CNN Approaches Relevant to Nanozyme-Based Biosensing

**Figure 3:**
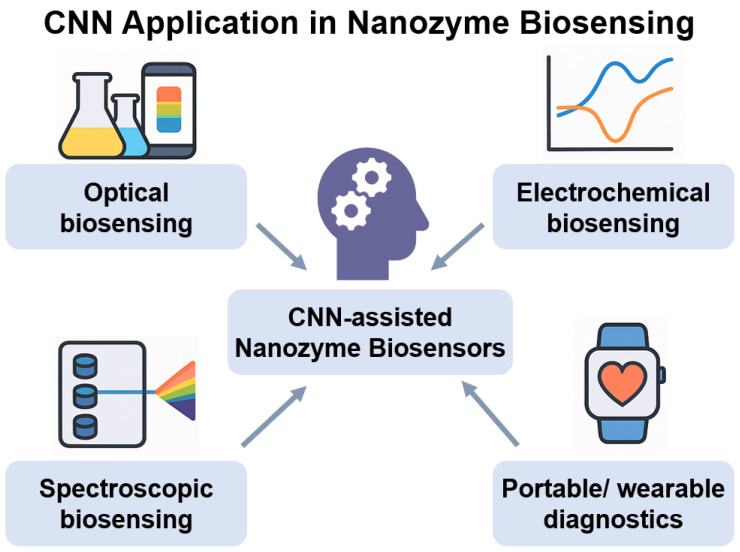
Schematic of CNN applications in nanozyme-based biosensing. CNNs enable automated analysis of complex biosensing outputs such as spectroscopic signals, imaging data, and voltametric curves, thereby improving detection accuracy, reducing interpretation time, and supporting real-time decision-making. Partially generated with ChatGPT (OpenAI, 2025).

The integration of CNNs with nanozyme-based sensing has transformed the interpretation of complex biosensing data by enabling automated feature extraction, signal classification, and real-time decision-making. CNNs are particularly effective at processing high-dimensional data, such as images, voltammograms, and spectral profiles, routinely generated by nanozyme-enabled biosensing platforms (Figure 3).

CNNs have been widely applied in optical and spectroscopic biosensing, including surface plasmon resonance (SPR) imaging and Raman spectroscopy. In SPR imaging, CNNs can detect subtle refractive index changes to quantify biomolecular interactions and classify cell types based on binding kinetics [119,120,121]. In Raman-based biosensing platforms—particularly those employing surface-enhanced Raman scattering (SERS)—CNNs effectively analyze complex spectral data by deconvolving overlapping peaks and identifying subtle vibrational shifts [122,123]. Although most current SERS biosensors rely on noble metal substrates, nanozymes have been integrated into SERS-active systems to enhance catalytic signal amplification and molecular specificity. While direct applications of CNNs to nanozyme-SERS platforms remain limited, their demonstrated success in conventional SERS analysis highlights strong potential for future integration in nanozyme-based spectroscopic diagnostics [5,105,106].

In electrochemical biosensing, CNNs facilitate the analysis of current-time curves, cyclic voltammograms, and impedance spectra, which are often complex and noisy [124,125,126,127]. Manual interpretation of these signals is challenging due to overlapping peaks and subtle variations. CNNs can reliably extract critical features such as peak shape, onset potential, and redox pair separation—parameters essential for quantifying biomarkers and detecting toxic metal ions. For instance, CNNs trained on voltammetric data can identify the distinct redox signature of dopamine in complex biological matrices, thereby improving detection sensitivity and specificity. Beyond these general capabilities, several representative studies demonstrate the practical use of CNNs in electrochemical biosensing. Fu et al. applied a one-dimensional CNN (1D-CNN) model to classify and quantify meat adulteration based on voltammetric signals, achieving high accuracy in discriminating subtle redox responses in complex food matrices [124]. Gecgel et al. employed DL to enhance the selective electrochemical detection of SARS-CoV-2, where CNN-based feature extraction improved sensitivity and specificity over manual interpretation [125]. More recently, Hoar et al. leveraged CNNs to deconvolute cyclic voltammograms with overlapping redox events, enabling reliable identification of redox-active species and mechanistic pathways [128]. These examples highlight how CNNs can transform electrochemical data analysis from descriptive interpretation to robust, automated diagnostics.

Beyond conventional modalities, CNNs have also been applied to genomic and proteomic analyses relevant to biosensing applications [129,130,131,132,133]. While these uses may not directly involve nanozymes, they highlight the versatility of CNNs in processing sequence-based data. Tasks such as mutation detection, protein structure prediction, and disease classification based on gene or protein expression profiles benefit from CNNs’ capacity to identify subtle sequence motifs and high-order relationships.

Importantly, CNNs are well suited for integration with wearable and POC diagnostic platforms, where real-time data from nanozyme sensors are collected via smartphones and embedded devices. In such settings, CNNs process multisensor data streams—including optical, thermal, and electrical signals—and deliver rapid, automated diagnoses without the need for expert interpretation. This capability supports continuous health monitoring, early disease detection, and personalized medicine, even in resource-limited environments.

Collectively, CNNs endow nanozyme-based biosensors with enhanced signal resolution, automated decision-making, and scalable analytical capabilities, laying the groundwork for next-generation intelligent diagnostic systems. Compared to these well-established AI-biosensor pairings, DL-nanozyme integration offers both complementary strengths and distinct challenges. Optical biosensors augmented by DL, such as SPR or SERS platforms, achieve exceptional sensitivity and molecular specificity but often rely on noble metals or sophisticated optics that limit cost-effectiveness and portability. Electrochemical biosensors combined with DL provide scalable, real-time signal analysis and are readily compatible with wearable formats, yet they remain susceptible to matrix interference and electrode fouling. In contrast, DL-nanozyme-based biosensors leverage the intrinsic catalytic robustness, tunability, and low-cost synthesis of nanozymes, enabling highly portable, multiplexed, and smartphone-integrated diagnostics. The main trade-off is maturity: nanozyme systems face greater challenges in synthesis reproducibility, long-term biocompatibility, and standardization compared to their optical and electrochemical counterparts. Nevertheless, the synergy between catalytic amplification and AI-driven interpretation positions DL-nanozyme biosensors as a distinctive and potentially transformative complement within the broader landscape of AI-enhanced diagnostics.

## 4. Practical Applications of DL-Enabled Nanozyme-Based Optical Biosensors

### 4.1. Synergistic Opportunities

The integration of DL with nanozyme-based biosensors represents a transformative approach to intelligent diagnostics. DL models—particularly CNNs and object detection frameworks such as You Only Look Once (YOLO)—can be applied to nanozyme sensor data for automated feature extraction, signal classification, and quantitative analysis. This synergy enhances detection sensitivity and specificity while aligning with the World Health Organization’s ASSURED criteria for POC diagnostics. Notably, DL enables real-time data interpretation, multiplexed analyte recognition, and adaptive decision-making—capabilities that conventional analytical methods often lack. Additionally, DL can assist in predicting catalytic parameters (e.g., *K*_m_, *V*_max_), optimizing nanozyme composition, and accelerating the design and deployment of biosensors.

Indeed, one of the critical advantages of integrating DL with nanozyme-based biosensing lies in its ability to mitigate long-standing technical challenges. Variability in signal responses—arising from batch-to-batch inconsistencies or environmental fluctuations—can be reduced through DL-enabled feature extraction and denoising, thereby improving reproducibility and standardization in nanozyme performance [134]. Moreover, multiplexed detection, often limited by overlapping signals and cross-reactivity, can be enhanced by DL-powered sensor arrays and object detection models. For instance, YOLO-assisted discrimination of multiple organophosphorus pesticides [135], CNN-based deconvolution of overlapping Raman peaks [136], and DL-enabled deconvolution of multichannel nanozyme readouts such as g-C_3_N_4_-based absorbance dynamics [137] have demonstrated how DL improves both sensitivity and robustness. Collectively, these examples illustrate that DL not only accelerates the optimization of nanozymes but also addresses persistent barriers in biosensing performance.

### 4.2. Smartphone-Integrated DL-Enhanced Nanozyme-Based Biosensors

The integration of DL and nanozyme-based biosensors has enabled the development of intelligent, portable, and low-cost diagnostic systems capable of automated signal interpretation. Among these systems, smartphone-assisted biosensors have emerged as highly accessible platforms, leveraging the ubiquity of mobile devices and their ability to perform real-time image acquisition and analysis.

This section highlights representative applications in which DL algorithms, such as CNNs, YOLO, MobileNet, and deep neural networks (DNNs), have been combined with nanozyme-catalyzed sensing mechanisms to support semi-automated detection of biologically and environmentally relevant analytes. These examples span colorimetric detection using red-green-blue (RGB)/hue-saturation-value (HSV) models (Section 4.2.1), multi-analyte discrimination via sensor arrays (Section 4.2.2), dual-mode platforms incorporating both colorimetric and fluorescent outputs (Section 4.2.3), mechanistic detection with multifunctional nanozymes (Section 4.2.4), and applications of broader ML architectures for regression and mixture analysis (Section 4.2.5).

Collectively, these platforms demonstrate capabilities for on-site, rapid, multiplexed, and real-time detection, depending on the sensing modality and DL model employed.

#### 4.2.1. DL-Powered Colorimetric Biosensors

Smartphone-integrated colorimetric biosensors powered by DL have shown considerable promise for portable, sensitive, and real-time diagnostics. As summarized in Table 3, these platforms integrate nanozyme-catalyzed chromogenic reactions with smartphone-based image acquisition and DL-driven data analysis. Representative applications target a wide range of analytes, including neurotransmitters, pesticides, and heavy metals, underscoring the versatility and broad diagnostic potential of this approach.

**Figure 4:**
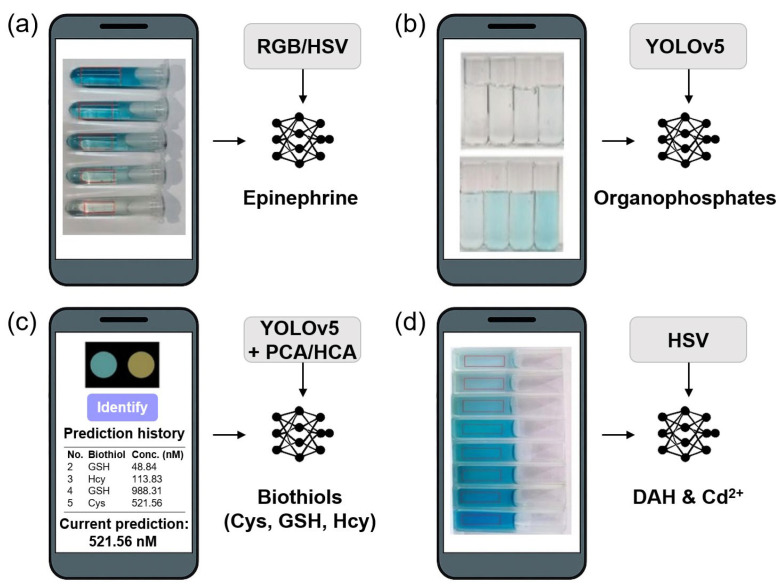
Smartphone-integrated DL-powered nanozyme colorimetric biosensors. (**a**) RGB/HSV-based quantification of epinephrine using La_0.96_Sr_0.04_NiO_3−δ_ nanozyme. Reprinted with permission from Ref. [138]. Copyright (2025) Elsevier B.V. (**b**) YOLOv5-assisted segmentation for organophosphate pesticides (glyphosate, paraoxon, omethoate) using a C_60_@MOF-545-Fe system. Reprinted with permission from Ref. [16]. Copyright (2025) Wiley-VCH GmbH. (**c**) Biothiol (Cys, GSH, Hcy) detection using CuZn-N SAzyme with YOLOv5 and PCA/HCA analysis via a smartphone app. Reprinted with permission from Ref. [12]. Copyright (2025) American Chemical Society. (**d**) Dual detection of DAH and Cd^2+^ using SrCoNiO_3−δ_ nanozyme analyzed through HSV-based regression. Reprinted with permission from Ref. [139]. Copyright (2025) Elsevier B.V.

Figure 4 shows representative smartphone-based DL-powered nanozyme colorimetric biosensors developed for real-time, on-site detection of diverse analytes, including neurotransmitters, pesticides, biothiols, and heavy metals. These platforms combine distinct nanozyme compositions and catalytic mechanisms with DL strategies such as RGB-based regression, clustering (PCA/HCA), and YOLO-driven segmentation, highlighting the adaptability of DL-nanozyme integration across biomedical and environmental sensing applications.

A representative approach employs perovskite-based La_0.96_Sr_0.04_NiO_3−δ_ nanozymes, in which strontium doping introduces OVs that enhance catalytic activity for epinephrine detection [138]. It is worth mentioning that the incorporation of DL substantially improved quantitative sensing performance relative to traditional analytical approaches. The DL-powered smartphone application enabled real-time analysis of colorimetric signals in the RGB and HSV domains, achieving excellent linear correlation (*R*^2^ ≈ 0.99) and a low detection limit of 0.1821 μM, confirmed in both buffer and biological matrices. Compared to conventional colorimetric or voltammetric sensors for epinephrine, the DL-assisted platform demonstrated a comparable or superior limit of detection, while offering greater convenience and reduced operator bias due to automated, algorithm-driven interpretation. Moreover, the custom DL model allowed for robust performance under diverse ambient lighting and image acquisition conditions, enhancing field applicability and reliability—factors that often limit conventional methods. However, it should be noted that the improvements in analytical outcome were most significant when complex sample backgrounds or variable imaging environments posed challenges to manual or classical algorithm analysis. For straightforward, high-contrast laboratory measurements, the relative benefit of DL integration over standard regression or thresholding may be modest. Thus, for this application, the use of DL was clearly justified for advancing portable and automated epinephrine biosensing, particularly where environmental variability or non-expert operation are critical considerations.

A notable advancement in enzyme-nanozyme cascade systems for POC diagnostics involved the development of a continuous flow reactor integrated with DL for multiplexed detection of organophosphorus pesticides (OPs) [16]. This system employed a C_60_@MOF-545-Fe nanozyme, in which oxidase- and peroxidase-like activities were significantly enhanced via host-guest interactions with fullerene (C_60_). Coupled with acetylcholinesterase (AChE), the reactor facilitated efficient cascade catalysis. A YOLOv5-based DL model was deployed on a smartphone platform for image segmentation and quantitative analysis, enabling automated identification, quantification, and differentiation of three OP analytes (glyphosate, omethoate, paraoxon) directly from colorimetric array images in field-relevant conditions. Quantitatively, the platform achieved low detection limits (glyphosate: 0.65 ng/mL, omethoate: 0.12 ng/mL, paraoxon: 0.32 ng/mL), broad linear ranges, and high selectivity and reproducibility in complex matrices, as validated in spiked real samples (soybean, rice, and apple) with recoveries typically between 97–104% and RSDs below 4%. Critically, DL was essential for accurate pattern recognition and multivariate regression within RGB/HSV color feature space, outperforming conventional visual or single-channel thresholding strategies—which are impractical for rapid, multiplexed, and user-independent assessments. The YOLOv5 model’s robustness to imaging variation (e.g., lighting, tube positioning) enabled reliable operation without laboratory-standard equipment or technical expertise, representing a clear advantage for POC deployment. Nevertheless, the benefits were most pronounced in multiplex and ambiguous scenarios; for single-OP, highly contrasted systems, conventional analysis may suffice. Thus, in this application, DL was unequivocally justified by the notable improvements in accuracy, usability, and throughput for rapid, on-site pesticide screening.

Further expanding the scope of detection, bimetallic SANs (CuZn-N SAzymes) have been used for the colorimetric sensing of biologically relevant thiol compounds, including cysteine, glutathione, and homocysteine. Here, DL frameworks such as YOLOv5, PCA, and HCA are embedded in a mobile application (“ThiolSense”) to enable accurate quantification in serum samples, reaching detection limits in the nanomolar range [12]. The system leveraged the YOLOv5 algorithm for automated segmentation and extraction of colorimetric data from smartphone-acquired images, followed by PCA and HCA for robust pattern recognition. This approach enabled accurate and multiplexed identification of cysteine, glutathione, and homocysteine with detection limits in the 1.17–1.35 nM range and a broad linear range down to clinically relevant concentrations. Critically, DL facilitated the real-time, user-independent processing of large, high-dimensional datasets, substantially decreasing susceptibility to variations in ambient lighting, imaging angle, and user operation—factors that commonly confound manual or traditional single-variable analyses. The portable “ThiolSense” app, powered by DL, enabled on-site quantification and mixture discrimination in both controlled and complex biological matrices (serum), confirming its clinical applicability by achieving high recoveries and low RSDs in actual sample analysis. Notably, while the incremental benefit of DL over conventional statistical discrimination may be less apparent for simple, well-separated colorimetric responses, its impact becomes pronounced when handling overlapping signals, complex mixtures, or field scenarios demanding reproducibility and throughput. Thus, in this context, DL was essential to advancing the sensor from a laboratory prototype to a practical, scalable POC diagnostic tool, greatly enhancing accuracy, selectivity, and operational robustness.

In the context of environmental monitoring, a SrCoNiO_3−δ_ trimetallic perovskite nanozyme was employed for the dual detection of dopamine hydrochloride (DAH) and cadmium ions (Cd^2+^) [139]. Synthesized via high-temperature calcination, the nanozyme’s peroxidase-like activity was significantly enhanced by the strategic substitution of Co^2+^ with Ni^2+^, which promoted electron transfer and facilitated the generation of OVs and ‧OH. The detection mechanism utilized a distinctive “on-off-on” colorimetric response: DAH induced a visible color change, which was subsequently reversed by Cd^2+^. Quantitative interpretation was achieved using a smartphone-based analytical platform powered by DL algorithms, incorporating RGB and HSV color space analysis. The system exhibited excellent analytical performance, with a linear detection range of 2–20 μM for both analytes and detection limits of 0.098 μM for DAH and 0.343 μM for Cd^2+^. Notably, the platform demonstrated strong reproducibility when applied to complex, field-relevant environmental samples. Furthermore, recovery studies in real samples, including soil and river water, confirmed detection precision with relative standard deviations below 5% and recovery rates from 97.27% to 106.25%. This work highlights the promising integration of advanced nanozyme catalysis with DL-enhanced smartphone sensing for practical environmental and biomedical applications, combining high sensitivity, reproducibility, and user-friendly operation in complex matrices.

Overall, a critical evaluation of DL applications in nanozyme-based biosensing, as compiled in Table 3, reveals significant benefits alongside notable challenges. The employed DL models have demonstrated superior capabilities in extracting complex features from high-dimensional, noisy data, enabling enhanced sensitivity, specificity, and multiplexed analyte recognition that traditional methods often cannot achieve. These improvements have translated into user-friendly, real-time diagnostic platforms, especially when integrated with smartphone-based detection, aligning with ASSURED criteria for POC applications. However, the implementation of DL also presents challenges that must be addressed to fully realize its potential. These include the scarcity of large, diverse, and standardized datasets crucial for robust model training and validation, variability in sample matrices and device hardware that introduce noise and affect reproducibility, and the need for lightweight, interpretable, and energy-efficient models suitable for portable deployment. Moreover, issues related to model transparency and clinical acceptance remain. Therefore, while DL integration has proven valuable and often worth the effort in advancing nanozyme-based biosensing performance, ongoing efforts focused on dataset standardization, model interpretability, and deployment robustness are essential for broader practical adoption and impact.

#### 4.2.2. Sensor Arrays for Multi-Analyte Discrimination

A DL-enhanced colorimetric sensor array (CSA) effectively discriminates structurally similar analytes, enabling both qualitative classification and quantitative analysis. These systems leverage nanozyme-catalyzed reactions and multichannel signal patterns, which are interpreted through advanced image processing and ML algorithms embedded in smartphone platforms.

**Figure 5:**
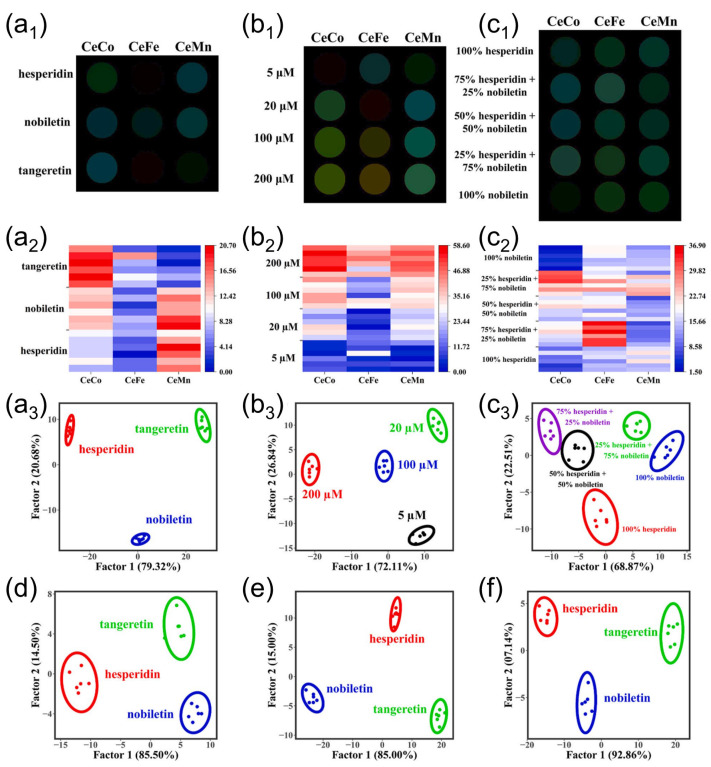
Representative illustrations of smartphone-assisted nanozyme-based CSA for flavonoid discrimination and quantification. (**a_1_**–**a_3_**) Difference maps, heat maps, and LDA plots for the classification of three flavonoids. (**b_1_**–**b_3_**) Concentration-dependent responses and discrimination. (**c_1_**–**c_3_**) Quantification of binary mixtures with varying ratios. (**d**–**f**) Robust classification performance in the presence of potential interferents. Reprinted with permission from Ref. [135]. Copyright (2024) Elsevier B.V.

A representative study reported a CeO_2_-based sensor array doped with Mn, Co, and Fe for the selective recognition of flavonoids such as hesperidin, nobiletin, and tangeretin [135]. Guided by density functional theory (DFT) predictions to optimize nanozyme activity, the researchers developed a three-channel array (CeMn, CeFe, CeCo) and quantified colorimetric responses using a segmentation-extraction-regression (SER-DL) workflow based on the MobileNetV3-small neural network. RGB image data were collected via a custom mobile application (“Quick Viewer”), enabling accurate flavonoid concentration predictions (*R*^2^ = 0.97). The system also achieved high classification performance, distinguishing flavonoids extracted from Citri Reticulatae Pericarpium samples stored for different durations using linear discriminant analysis (LDA). As illustrated in Figure 5 [135], the MobileNetV3-small model integrated with the smartphone-based sensor array enabled precise quantification and discrimination of structurally similar flavonoids via the SER-DL workflow. Difference maps and LDA plots further confirmed robust classification performance, even in mixtures of varying flavonoid ratios and in the presence of interferents, validating the platform’s applicability for real-sample analysis.

In a related study, a MnO_2_ nanozyme-based CSA was developed for the rapid, low-cost detection of unsaturated fatty acids, including oleic acid, linoleic acid, and α-linolenic acid [140]. The sensor emulated olfactory signal processing by converting competitive inhibition—induced colorimetric changes into analyte-specific response patterns. A DL-enhanced workflow was implemented using an image segmentation-feature extraction-regression (ISFE-DL) model based on MobileNetV3-small, trained on over 38,000 single-hole images. Quantitative analysis was conducted via a smartphone application (“Intelligent Analysis Master”), yielding high determination coefficients (*R*^2^ = 0.9969 for oleic acid, 0.9668 for linoleic acid, and 0.7393 for α-linolenic acid). Additionally, LDA facilitated mixture classification and multicomponent quantification, demonstrating the system’s potential for real-world compositional analysis.

Collectively, these examples illustrate how nanozyme-based CSAs, when integrated with lightweight DL models and mobile analytics, enable reliable, accessible, and multiplexed detection of complex molecular targets across food, biomedical, and environmental applications.

#### 4.2.3. Dual-Mode Detection and Smart Sensing

**Figure 6:**
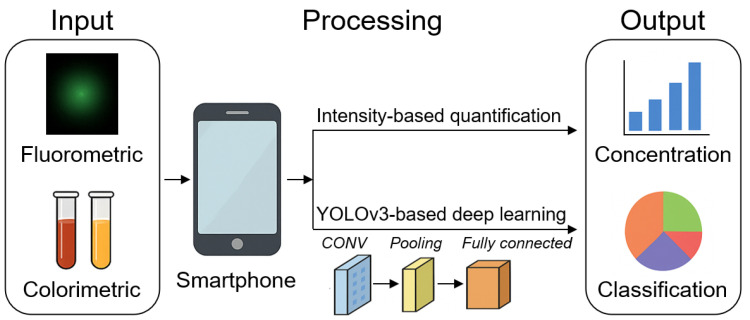
Schematic of dual-mode smartphone-integrated nanozyme-based biosensing system. Fluorometric signals are quantified through intensity-based quenching analysis, while colorimetric signals are processed by a YOLOv3-based DL pipeline (convolutional layer, pooling, and fully connected) to extract RGB/HSV features. The outputs include quantitative concentration values and classification results. CONV = Convolutional layer. Partially generated with ChatGPT (OpenAI, 2025).

Dual-mode nanozyme-based biosensors that combine colorimetric and fluorometric outputs provide enhanced reliability and analytical accuracy by mitigating limitations associated with single-mode detection. A representative example is the smartphone-integrated sensing system developed for detecting tetracycline analogs, including tetracycline, chlortetracycline, oxytetracycline, and doxycycline [141]. The system used a NH_2_-MIL-88 B (Fe, Ni) nanozyme exhibiting both peroxidase-like activity via Fenton-like reactions and intrinsic blue fluorescence. The detection mechanism leveraged dual-signal responses: the analogs inhibited the peroxidase-like activity, reducing the blue coloration of oxidized 3,3′,5,5′-tetramethylbenzidine (TMB), and simultaneously quenched fluorescence through an internal filtering effect. To facilitate real-time, portable analysis, the authors integrated the nanozyme with a three-dimensional (3D) printed smartphone imaging system and a custom WeChat applet (“Intelligent Fluorescence Analysis”). A YOLOv3-based DL model was employed for image recognition, facilitating RGB and HSV color feature extraction from both colorimetric and fluorescent images. This dual-mode platform achieved wide detection ranges with low detection limits—0.182 and 0.0668 μM for the colorimetric and fluorescent channels, respectively—while maintaining high specificity and minimal false-positive rates. Validation with real samples, including milk and water, confirmed the system’s reproducibility and stability. This approach exemplifies the synergistic integration of nanozyme catalysis, multimodal sensing, smartphone hardware, and DL analytics, offering a robust and versatile solution for POC antibiotic residue monitoring. An overview of this integrated detection strategy is presented in Figure 6, highlighting the smartphone-based dual-mode nanozyme-based biosensor enhanced by YOLOv3-driven analysis.

#### 4.2.4. Mechanistic Detection and Multifunctional Systems

The development of multifunctional nanozyme platforms that integrate high catalytic activity with biosensing and environmental remediation capabilities represents a promising direction in nanozyme research. A notable example involves Co_3_O_4_/CoFe_2_O_4_ hollow nanocubes (HNCs), designed with abundant OVs and a porous core-shell heterostructure comprising a Co_3_O_4_ core and CoFe_2_O_4_ shell [11]. Synthesized via a one-pot method, these HNCs exhibited peroxidase-, oxidase-, and catalase-like activities, supporting their potential for diverse applications.

Comprehensive characterization, including X-ray photoelectron spectroscopy depth profiling and DFT simulations, revealed that the high peroxidase-like activity of the HNCs stemmed from ‧OH generation, mediated by synergistic interactions between the surface and interior OVs and dynamic electron transfer between the Co and Fe centers.

To translate this multifunctionality into a real-time diagnostic tool, a smartphone-based sensing platform incorporating a YOLOv3 DL algorithm was developed. The system enabled in situ detection of diverse analytes such as L-cysteine, norfloxacin, and zearalenone. Among these analytes, norfloxacin was detected with high sensitivity and a detection limit of 0.015 μM, which is superior to that of many previously reported methods. In addition, the HNCs demonstrated exceptional performance in degrading rhodamine B (99.24% efficiency), with high reusability over 10 catalytic cycles, highlighting their dual function in biosensing and environmental cleanup.

Mechanistic validation was performed via in situ Fourier transform infrared (FTIR) spectroscopy. Spectral analysis confirmed the binding and oxidation sequence in the L-cysteine/norfloxacin detection pathway: the formation of S-M (metal) bonds with L-cysteine led to the inhibition of the nanozyme, which was subsequently reversed upon norfloxacin-induced oxidation, restoring catalytic activity and generating an “on-off-on” colorimetric signal.

**Figure 7:**
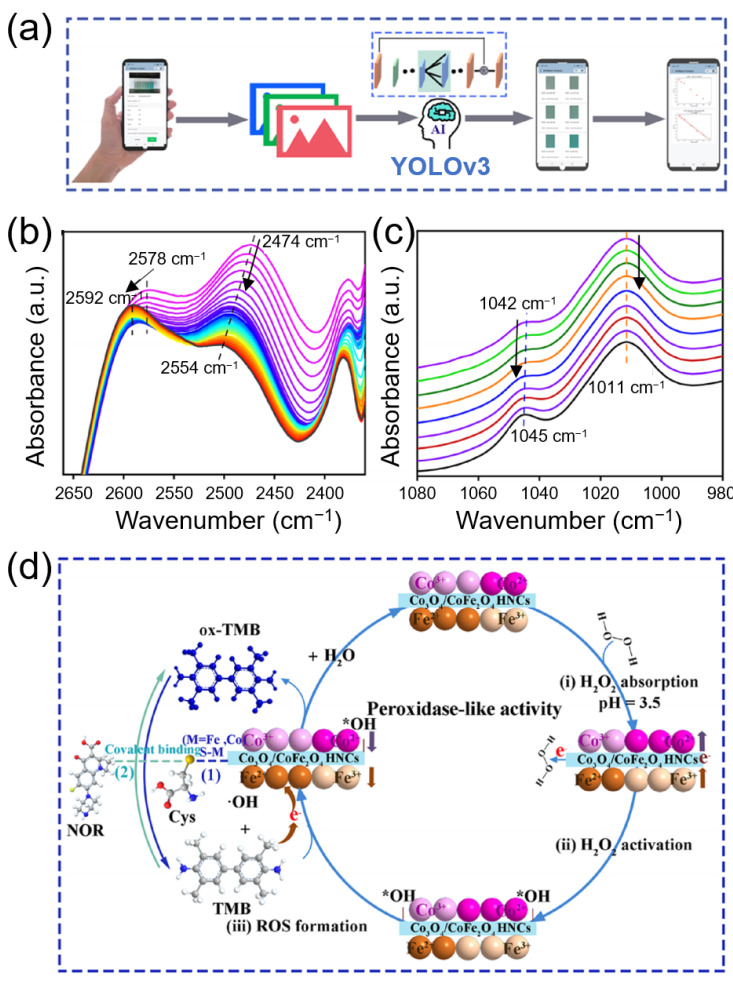
Illustration of YOLOv3-based DL-assisted smartphone biosensing system using Co_3_O_4_/CoFe_2_O_4_ HNCs. (**a**) Workflow of image acquisition, analysis, and concentration prediction using RGB/HSV features. (**b**) In situ FTIR spectra showing the decrease in the –SH stretching band during cysteine (Cys) binding to the nanozyme surface, indicating inhibition of electron transfer. (**c**) Subsequent FTIR spectra illustrating norfloxacin (NOR)-induced oxidation of cysteine and the emergence of S=O stretching, confirming recovery of electron transfer in the “on-off-on” mechanism. (**d**) Proposed catalytic and signal generation mechanism involving ‧OH production and reversible electron transfer through S–M bond formation and norfloxacin-mediated recovery. Reprinted with permission from Ref. [11]. Copyright (2023) American Chemical Society. *OH denotes a hydroxyl species adsorbed on the catalyst surface, serving as a key reaction.

The full workflow of the intelligent biosensing platform and the underlying catalytic mechanism are illustrated in Figure 7. The DL-assisted detection process (Figure 7a) employed YOLOv3 for image segmentation and RGB/HSV-based concentration prediction via a WeChat mini app. Mechanistic validation using in situ FTIR (Figure 7b,c) confirmed that L-cysteine binds to the nanozyme surface through S–M (metal) bonds, inhibiting electron transfer and suppressing TMB oxidation. Upon addition of norfloxacin, *L*-cysteine undergoes oxidation, restoring electron transfer and enabling colorimetric signal recovery. This “on-off-on” response mechanism (Figure 7d) exemplifies the synergistic integration of molecular interaction analysis and DL in multifunctional nanozyme-based biosensing.

This work highlights how advanced nanozyme platforms can integrate DL analytics and molecular-level mechanistic understanding to support next-generation, multifunctional biosensing systems.

#### 4.2.5. Expansion to General ML Architectures

Although CNNs and object detection frameworks like YOLO dominate the landscape of DL-powered biosensors, broader ML architectures also contribute significantly to nanozyme-based sensing, particularly for tasks such as mixture discrimination and quantitative regression. A representative example features a gold nanozyme-based CSA designed for the classification and quantification of monosaccharides [142]. This system mimicked glucose oxidase-like activity by employing multiple electron acceptors—O_2_, 2,2′-azino-bis(3-ethylbenzothiazoline-6-sulfonic acid ammonium salt) (ABTS)^+∙^, and [Ag(NH_3_)_2_]^+^—to oxidize various monosaccharides, generating distinct cross-reactive responses across three detection channels.

Colorimetric data from the array were analyzed using classical ML algorithms, specifically LDA and hierarchical clustering analysis (HCA), to classify mixtures of D-glucose and D-fructose at varying ratio gradients (25%, 10%, and 5%). The resulting 2D canonical score plots and confusion matrices demonstrated high classification accuracy, although precision decreased slightly at lower gradient differences. For simultaneous quantification, a DNN-based regression model was constructed, incorporating multiple hidden layers with tailored activation functions and learning parameters. This model accurately predicted the concentrations of glucose and fructose in mixed samples, as evidenced by regression plots showing narrow error margins and strong concordance with high-performance liquid chromatography results.

**Figure 8:**
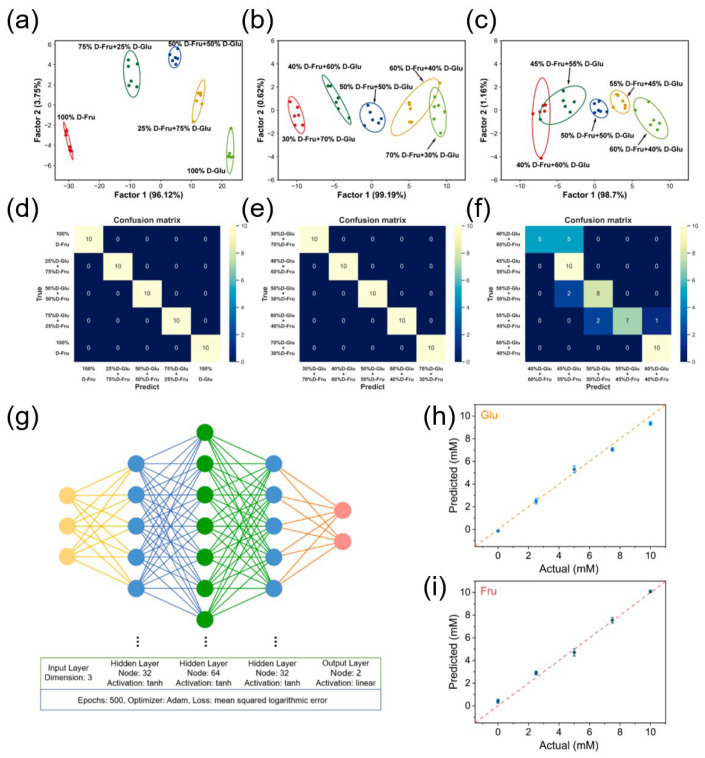
Visualization of ML-assisted analysis of Au nanozyme-based sensor array responses for monosaccharide mixtures. (**a**–**c**) 2D canonical score plots from LDA for classifying glucose and fructose mixtures at 25%, 10%, and 5% gradient differences, respectively. (**d**–**f**) Corresponding confusion matrices highlight classification accuracy. (**g**) DNN regression model architecture used for concentration prediction. (**h**,**i**) Regression plots show predicted versus actual glucose and fructose concentrations in mixtures, demonstrating high accuracy. Reprinted with permission from Ref. [142]. Copyright (2024) Elsevier B.V.

Overall, this study exemplifies how ML algorithms beyond conventional CNNs can be effectively harnessed in nanozyme-based biosensing for multi-analyte classification and compositional analysis. The integration of ML models with nanozyme-based sensor arrays offers a flexible and scalable approach for decoding complex biochemical mixtures with high sensitivity and interpretability. As illustrated in Figure 8, LDA enables accurate classification, while DNNs support precise regression across glucose-fructose mixtures at varying ratios (25%, 10%, and 5%). The architecture and performance of the DNN model underscore its strong predictive capability, yielding concentration estimates with minimal error (Figure 8g–i). This convergence of DL and nanozyme-based biosensing is paving the way for a new generation of intelligent, portable, and cost-effective diagnostic platforms with broad applicability—from neurotransmitter and toxin detection to food safety and environmental monitoring.

When viewed within the broader biosensing and AI-driven diagnostics landscape, DL-nanozyme biosensors share similarities with other AI-integrated approaches while also offering distinctive features. DL-optical biosensors, for instance, benefit from high-resolution imaging pipelines but often face challenges such as photobleaching and limited robustness under field conditions. DL-electrochemical biosensors provide highly sensitive and low-cost detection, yet issues such as electrode fouling and complex calibration can limit reproducibility. By comparison, DL-nanozyme biosensors leverage catalytic amplification, stability under diverse conditions, and cost-effectiveness, while still facing trade-offs including batch-to-batch variability and biocompatibility concerns. This comparative synthesis situates DL-nanozyme integration within the broader AI-biosensor landscape, clarifying both its distinctive opportunities and remaining challenges.

## 5. Challenges and Future Perspectives

The future of AI-enhanced nanozyme-based biosensing depends on overcoming several interconnected obstacles. For clarity, these can be prioritized into four tiers, each paired with research opportunities that represent the most logical next steps:

### 5.1. Most Pressing: Data Standardization and Model Generalizability

The lack of high-quality, standardized biosensing datasets is the single greatest bottleneck, as it directly limits reproducibility and cross-platform deployment. Future direction: creation of open, annotated biosensor databases and adoption of federated learning frameworks to improve generalizability while protecting data privacy.

### 5.2. Model Interpretability and Clinical Trust

Although DL offers powerful predictive capabilities, its “black box” nature poses challenges for adoption in biosensing and clinical diagnostics. For nanozyme-based sensors, interpretability is particularly critical because clinicians and end-users must understand how sensor outputs are generated from complex raw signals (e.g., colorimetric, electrochemical, or spectroscopic data). Lack of transparency can undermine trust in POC devices, even when analytical accuracy is high. Therefore, model-agnostic explainability tools (e.g., SHAP, Grad-CAM) and inherently interpretable architectures should be integrated to clarify how features are extracted and decisions are made. Such approaches are especially important for safety-critical applications—including cancer biomarker detection, infectious disease screening, and environmental monitoring—where reliable sensor readouts directly impact decision-making.

### 5.3. Technical Integration: Real-Time Deployment and IoT Compatibility

Variability in smartphones, calibration issues, and environmental noise complicate field use. Future direction: optimization of lightweight edge-AI models and standardized calibration protocols to ensure robustness under real-world conditions, especially for Internet of Medical Things (IoMT)-enabled platforms.

### 5.4. Matrix Effects and Real-Sample Validation

Although DL-enhanced nanozyme biosensors show excellent performance in standard solutions, their translation to real biological or environmental samples poses additional challenges. Complex sample matrices (e.g., blood, serum, milk, wastewater) can introduce matrix effects such as nonspecific adsorption, autofluorescence, and interfering ions, which reduce catalytic activity and signal fidelity. Furthermore, variability among real samples often undermines the robustness of DL models trained on controlled datasets. Addressing these issues will require improved surface passivation of nanozymes, rigorous validation in clinically relevant matrices, and the integration of adaptive or transfer learning strategies to maintain model accuracy across heterogeneous real-world conditions.

### 5.5. Long-Term Materials Challenge: Synthetic Control and Biocompatibility

Batch-to-batch variability and unresolved safety concerns remain barriers to clinical translation. Future direction: advances in atomic-level control (e.g., SANs/DANs) and surface modification, along with rigorous in vivo safety and biodegradability studies, will be essential for regulatory approval.

Taken together, this tiered framework highlights a progression from urgent, short-term challenges (data availability and model trustworthiness) to medium-term integration hurdles (real-world deployment and IoMT compatibility) and finally to long-term translational issues (synthetic reproducibility and biocompatibility). This prioritization not only clarifies where research investment is most critical but also outlines a coherent roadmap: establish reliable data infrastructure, ensure interpretability and clinical trust, achieve robust deployment in mobile and IoT systems, and ultimately secure safe, reproducible materials for clinical translation.

Beyond these technical and scientific priorities, it is essential to anchor DL-nanozyme-based biosensing within the broader realities of 2025 research and commercialization. The COVID-19 pandemic accelerated demand for decentralized and mobile diagnostics, catalyzing the widespread integration of AI with biosensors across infectious disease, oncology, and metabolic health. Recent advances in smartphone- and IoMT-enabled biosensing already demonstrate how DL can deliver automated, low-cost, and user-friendly diagnostics in both field and home settings. At the same time, major funding initiatives—including NIH programs on AI for precision health, Horizon Europe calls for AI in medical diagnostics, and Korea’s NRF programs on AI-driven biosensors—reflect strong global investment in this space. Commercially, start-ups and established companies are translating AI-enhanced biosensors into POC devices for glucose monitoring, cardiovascular risk assessment, and infectious disease screening, underscoring the momentum toward real-world deployment. Against this backdrop, DL-nanozyme biosensors occupy a strategically important niche: their catalytic robustness supports affordable and portable testing, while their compatibility with AI analytics enables intelligent, networked healthcare solutions. This convergence demonstrates not only technological feasibility but also clear translational potential in the near term.

## 6. Conclusions

The convergence of DL and nanozyme-based biosensing has initiated a transformative shift in the development of intelligent, portable, and highly sensitive diagnostic platforms. By harnessing the unique catalytic properties of nanozymes alongside the robust pattern recognition and feature extraction capabilities of DL models, researchers have enabled smartphone-integrated systems capable of real-time, on-site, and multiplexed detection across diverse biomedical and environmental targets.

Recent advances have enabled the integration of DL algorithms—such as CNNs, YOLO architectures, and MobileNet variants—with colorimetric, electrochemical, and spectroscopic sensing modalities. These DL-enhanced platforms offer automated signal interpretation, high-throughput analysis, and minimal user input, aligning with the demands of POC and resource-constrained diagnostics. Moreover, innovations such as dual-mode detection, sensor arrays, and multifunctional nanozymes have significantly broadened the versatility and application scope of these systems.

However, critical challenges remain, including data standardization, model interpretability, deployment robustness in real-world settings, and the biocompatibility of nanozymes. Overcoming these limitations will require close interdisciplinary collaboration among materials scientists, chemists, computer scientists, and clinicians.

The future of DL-enhanced nanozyme-based biosensors is highly promising. Emerging advances in federated learning, IoMT integration, sustainable nanomaterial development, and personalized analytics are expected to drive the development of next-generation diagnostic platforms that are accurate, scalable, adaptive, and broadly accessible.

## Figures and Tables

**Table 1:** Classification of nanozymes by composition and catalytic activity.

Category	Subtypes/Examples	Typical Enzyme Mimicry	Key Features	Representative Applications
Metal/Metal Oxide	Fe_3_O_4_, MnO_2_, CeO_2_, Au, Pt, Pd	Peroxidase, Oxidase, Catalase	High catalytic activity, redox cycling, robust in harsh media	Colorimetric biosensing, ROS modulation
Carbon-Based	Graphene, GO *, CNTs *, CQDs *, g-C_3_N_4_	Peroxidase, Oxidase	Tunable surface chemistry, high conductivity	Electrochemical sensing, antibacterial agents
MOF/COF-Based	MOF-545 *, ZIF-8, COF *-TpPa	Peroxidase, Oxidase	High porosity, modular structure, controllable active sites	Multiplex biosensing, environmental sensing
Polymer/Organic	Imidazole-functionalized polymers, micelles	Oxidase, Peroxidase	Biomimetic microenvironment, low toxicity	In vivo detection, drug delivery
Single-/Dual-Atom Nanozymes	Fe-N_4_, Cu-N, Fe-Fe SANs*/DANs	Peroxidase, Catalase	Maximum atom utilization, high selectivity	Precision biosensors, enzyme replacement

* Abbreviations: GO, graphene oxide; CNTs, carbon nanotubes; CQDs, carbon quantum dots; MOF, metal-organic framework; COF, covalent organic framework; SANs, single-atom nanozymes; DANs, dual-atom nanozymes.

**Table 2:** Active site engineering strategies for nanozymes.

Strategy	Mechanism/Target	Effects on Catalysis	Representative Materials	Applications
Surface Doping/ Elemental Substitution	Introduction of heteroatoms (e.g., N, P, and S) into carbon or metal oxide lattices	Modifies electronic structure; creates or enhances active sites; tunes d-band center	N-doped carbon dots; S-doped CeO_2_; P-doped MnO_2_	ORR/OER catalysis, ROS scavenging, biosensing
Defect Engineering	Generation of OVs, edge defects, and dislocations	Provides unsaturated coordination sites; enhances electron transfer and adsorption	Oxygen-deficient CeO_2_, MoS_2_ edge-defected nanosheets	Peroxidase-like catalysis, H_2_O_2_ sensing
Facet Exposure Control	Synthesis-directed exposure of specific crystal planes (e.g., {111}, {100})	Alters surface atom arrangement; modulates substrate binding and reaction pathways	Fe_3_O_4_ (111), CeO_2_ (100)	Selective biomarker detection, ROS modulation
Single-/Dual-Atom Dispersion	Isolated atoms or atom pairs anchored on supports (e.g., Fe-N_4_, Cu-N, Fe-Fe)	Maximizes atom utilization; unique reactivity due to quantum and support interactions	SANs on graphene, Pt-Fe DANs on N-doped carbon	Enzyme-mimetic biosensors, drug monitoring
Surface Functionalization	Attachment of ligands, polymers, antibodies, or aptamers	Improves biocompatibility; enhances substrate specificity and matrix stability	Ab-functionalized Au nanozymes, PEGylated Fe_3_O_4_	Targeted diagnostics, in vivo sensing, immunoassays

**Table 3:** Summary of DL-powered colorimetric nanozyme-based biosensors.

Target Analyte	Nanozyme Type	DL Architecture	Smartphone Function	LOD	Notable Features	Ref
Epinephrine	La_0.96_Sr_0.04_NiO_3−δ_ (perovskite)	Custom DL with RGB/HSV analysis	Real-time quantification via RGB/HSV image analysis	0.1821 μM	High linearity (*R*^2^ > 0.99); multi-color model use	[138]
Glyphosate, Omethoate, Paraoxon (OPs)	C_60_@MOF-545-Fe + AChE cascade	YOLOv5-OPs	Image segmentation, RGB/HSV feature extraction, linear fitting	Not explicitly listed; multiplex confirmed	Multiplexed OP detection; YOLOv5 WeChat app	[16]
Cys, GSH, Hcy (biothiols)	CuZn-N bimetallic SAzyme	YOLOv5 + PCA *, HCA *	“ThiolSense” app; RGB data + clustering	1.17 nM	Dual-channel array; serum quantification	[12]
Dopamine hydrochloride (DAH), Cd^2+^	SrCo NiO_3−δ_ (perovskite)	HSV/RGB + DL regression	On-off-on color change detection + app-based regression	DAH: 0.098 μM Cd^2+^: 0.343 μM	Environmental sensing; on-site detection	[139]

* Abbreviations: PCA, principal component analysis; HCA, hierarchical clustering analysis.

## Data Availability

No new data were created.

## Abbreviations

The following abbreviations are used in this manuscript:

ABTS	2,2’-azino-bis(3-ethylbenzothiazoline-6-sulfonic acid ammonium salt)
AI	artificial intelligence
ASSURED	Affordable, Sensitive, Specific, User-friendly, Rapid and robust, Equipment-free, and Deliverable
CNN	convolutional neural network
COF	covalent organic framework
CQD	carbon quantum dot
CSAs	colorimetric sensor arrays
DAN	dual-atom nanozyme
DFT	density functional theory
DL	deep learning
DNN	deep neural network
FTIR	Fourier transform infrared spectroscopy
GO	graphene oxide
HCA	hierarchical clustering analysis
HNCs	hollow nanocubes
HSV	hue-saturation-value
IoMT	Internet of Medical Things
LDA	linear discriminant analysis
ML	machine learning
MOF	metal-organic framework
OER	oxygen evolution reaction
OVs	oxygen vacancies
POC	point-of-care
RGB	red-green-blue
ROS	reactive oxygen species
SAN	single-atom nanozyme
SERS	surface-enhanced Raman scattering
SPR	surface plasmon resonance
TMB	3,3’,5,5’-tetramethylbenzidine
YOLO	You Only Look Once
